# Inherited glomerular diseases in the *gilded age* of genomic advancements

**DOI:** 10.1007/s00467-019-04266-y

**Published:** 2019-05-03

**Authors:** Ashima Gulati, Neera Dahl, Alda Tufro

**Affiliations:** 1 Department of Internal Medicine/ Nephrology, Yale University School of Medicine, New Haven, CT USA; 2 Department of Pediatrics/ Nephrology, Yale University School of Medicine, New Haven, CT USA; 3 Department of Cell and Molecular Physiology, Yale University School of Medicine, New Haven, CT USA

**Keywords:** Genetic glomerulopathies, Whole exome sequencing, Gene panels

## Abstract

The synchronized advent of high-throughput next-generation sequencing technology and knowledge of the human genome has rendered exponential contributions to our understanding of the pathophysiology of glomerular kidney diseases. A genetic diagnosis can now be made or confirmed in about two-thirds of the suspected inherited glomerular diseases. Next-generation sequencing is adept at identifying single nucleotide variations and small insertions or deletions that constitute majority of the disease-causing mutations. Description of the complete mutation spectrum in syndromic glomerulopathies may require the use of both sequencing and cytogenetic methods to detect large structural DNA variation in addition to single nucleotide changes. The enthusiastic application of genetic and genomic knowledge to inherited glomerular diseases has uncovered anticipated and unforeseen challenges mainly related to the biological interpretation of *variants of uncertain significance* and the limited benefit on clinical management for the individual patient when a diagnosis is obtained. To attain the ultimate goal of transforming clinical decision-making based on accurate genetic diagnosis using genomic information, these challenges need to be addressed. Till then, the glory of genomic medicine stands the test of time in this *gilded age* of genomic advancements.

## Introduction

Glomerular diseases constitute almost a quarter of etiologically defined end-stage renal disease (ESRD) in children (22%; similar to CAKUT), with focal segmental glomerulosclerosis (FSGS) being the most common individual diagnosis (12%) associated with pediatric ESRD in the 2017 USRDS annual data report [1]. The proportion of ESRD of unknown or unspecified cause is significant at 21% and steadily increases with age, ranging from 11% between 0 and 4 years of age up to 27% in the 18–21-year age group*.* Since glomerular conditions causing chronic kidney disease (CKD) are also more common with increasing age, it is expected that a significant proportion of glomerular disease remains etiologically unknown or misclassified.

Unsolved and misdiagnosed glomerular disease poses relevant clinical challenges. It is often classified as idiopathic, portrays substantial morbidity related to proteinuria and progressive CKD requiring long-term follow-up and care. These characteristics identify glomerular conditions as the most important group where genetic diagnosis made using recent genomic advancements in the field can be best utilized for maximum clinical advantage. An accurate molecular diagnosis can guide selection of treatment options to minimize toxicity, inform prognosis, and risk of post-transplant recurrence or familial disease. The 5-year kidney graft survival for children transplanted for all glomerular diseases combined is low at ~ 73% (NAPRTCS annual report 2014; [2]). Recognition of the underlying genetic cause where feasible would help stratify pre- and post-transplant risks, prevent inadvertent exposure to toxic therapies, and facilitate quest for specific therapies as pathophysiologic understandings advance. Patients with glomerular diseases also are frequently involved in clinical trials comparing therapies, which would benefit from including genotype-phenotype data in the response to treatment and outcome analysis.

The seemingly rapid evolution of available genetic and genomic testing methods for inherited glomerular diseases has provided the opportunity of offering a genetic diagnosis and has uncovered challenges of finding the preferred and feasible testing approach. Here, we discuss available genetic testing methodologies for glomerular diseases and use real clinical scenarios exemplifying the most applicable genetic testing approach.

## Genetic and genomic testing methods for inherited glomerular diseases

As genetic testing methodologies evolve and expand, it becomes imperative to select the most appropriate available option. While research testing protocols are guided by institutional review boards, the requirements for clinical genetic testing are uniformly regulated by Clinical Laboratory Improvements Amendments (CLIA) (https://www.cms.gov/Regulations-andGuidance/Legislation/CLIA). The Genetic Testing Registry is a resource listing available genetic testing options for various clinical conditions including some of the glomerular kidney diseases (GTR®; https://www.ncbi.nlm.nih.gov/gtr/). This database allows for voluntary submission of genetic test information by lab providers worldwide. A review of the GTR® data shows that currently, about 16,000 genes can be tested for over 10,000 conditions in about 500 labs worldwide. This is a striking change from 5 years ago when only 966 genes could be tested for 785 conditions in fewer than 100 labs in 2012–2013. Some of the available genetic testing methodologies listed in the GTR® include Karyotyping, FISH, CNV analysis, targeted variant analysis, sequence analysis of select exons, and sequence analysis or mutation scanning of the entire coding region of the genome. Next-generation sequencing is rapidly taking over as a widely available and used genetic testing methodology. Both conventional and molecular cytogenetic testing methods still maintain a complementary or supplementary role in specific situations. Genetic testing methods can broadly be categorized as *cytogenetic testing and sequencing methods.*

### Cytogenetic testing

Cytogenetics is the study of chromosomes made possible by light microscopic inspection of cells that are actively dividing [3]. Chromosomes numbered 1–22 according to their decreasing size differ in their centrosome position and banding pattern as stained by quinacrine (Q-banding) or Giemsa (G-banding) or R-banding (reverse pattern of G-bands). Peripheral blood lymphocytes are most suitable for cytogenetic analysis. Other actively dividing cell sources such as bone marrow and lymph nodes can also be used. Karyotype information or visual inspection of chromosomes reveals the total number of chromosomes in the cell, including the sex chromosome. Karyotypes can demonstrate chromosomal abnormalities such as chromosomal copy number variations (CNV) of gain (e.g., trisomy 21 or Down syndrome) or loss (e.g., loss of one X chromosome in phenotypic females with Turner syndrome) of an entire or part of a chromosome, as well as chromosomal structural rearrangements such as inversion of a chromosomal segment. Cytogenetic nomenclature has been standardized for uniform reporting [4, 5]. Karyotyping is an essential tool to confirm suspicion of Frasier syndrome resulting from Wilms tumor-suppressor gene 1 (*WT1*) mutation and characterized by steroid-resistant nephrotic syndrome and focal segmental glomerulosclerosis (FSGS) presentation in phenotypic females with a XY male karyotype who are at risk for gonadal tumors [6].

Molecular cytogenetic tests, including fluorescence in situ hybridization (FISH) and comparative genomic hybridization (CGH) also known as chromosomal microarray analysis (CMA), are a good adjunct to karyotyping and conventional cytogenetics [3]. These are hybridization assays that use fluorescent complementary DNA probes to detect or demonstrate the lack of a segment of DNA sequence and thus can detect CNVs that may represent an important component of the mutation spectrum of certain diseases especially for syndromic conditions. A karyotype, in addition to detecting syndromes of chromosomal loss or gain, can detect larger chromosomal structural aberrations up to a resolution of 5–10 Mb [5]. FISH uses probes targeted to detect the presence or absence of a specific DNA sequence up to a resolution of 100 Kb. An array-based comparative genomic hybridization (aCGH) uses many DNA probes to interrogate the entire genome for CNVs in a test sample and compares it to control samples. Similarly, a single nucleotide polymorphism (SNP)–based array uses SNP that are common single-base pair variations spread across the genome and present in > 1% of the general population, as makers to detect CNVs across the genome of a test sample and compares results to that of a known general population control database. An array CGH is a genome-wide approach that can identify CNVs up to 20–200 Kb across the entire genome and thus may be useful to identify aberrations in cases that are negative by karyotyping and FISH [7]. Thus, CGH, FISH, and karyotyping may be used in conjunction for identification of CNVs of varying magnitude, for localization of the exact chromosomal region, and for confirmation of results.

While the majority of disease-causing mutations include single nucleotide variants (SNV) or small insertion and deletions that can readily be identified by next-generation sequencing methods discussed below, description of the complete mutation spectrum in syndromic glomerulopathies may require use of both cytogenetic and sequencing methods to detect CNVs in addition to single nucleotide changes. For example, genetic investigation of a series of 20 patients with nail–patella syndrome using array CGH identified a 2 Mb deletion encompassing the entire *LMX1B* gene in a patient with a complex clinical phenotype in addition to the more commonly observed substitution mutations and small insertion/deletions that were identified by sequencing [8]. Similarly, the most common abnormality in cystinosis is a 57 Kb deletion in the *CTNS* gene that can be identified using FISH or array CGH [9].

Identification of large deletions by next-generation sequencing though possible, the sensitivity depends on the read depth of the region requiring additional vigilance and thus may merit additional communication with the clinical lab regarding such analysis [10]. This may be particularly useful in cases with syndromic or multi-systemic involvement where deletion of a genomic segment including the entire or more than one gene is suspected. European guidelines recommend validation of CNV in exome data by genome-wide array analysis or another suitable technique [5].

### Sequencing methods

Molecular genetic testing for single to few genes in their entirety, or for specific variants in a single gene, can be done using Sanger sequencing, polymerase chain reaction–based assays or using next-generation or high-throughput–targeted direct sequencing. Similarly, genome-scale sequencing can be done using next-generation or high-throughput direct sequencing that employs high-efficiency rapid DNA sequencing [11]. Sanger sequencing is still considered the gold standard approach for confirmation of individual gene variants identified through next-generation sequencing, although advances in the analysis techniques and use of robust quality scores for high-confidence variant calls have reduced the need for Sanger confirmation in many cases.

#### Limited gene panel–based testing

Testing for specific gene variants, e.g., in carrier or familial testing, single genes, or gene panel listing underlying genes responsible for specific monogenic diseases, is a commonly used approach for clinical testing by CLIA-certified labs. Such gene panels are based on current knowledge pertaining to a specific clinical diagnosis, clinical presentation, or phenotype. Gene panels are usually lab customized and hence differ between labs. This *limited gene panel–based testing* may utilize Sanger sequencing or next-generation sequencing technology targeted to a set of genes.

#### Whole exome and genome sequencing

Next-generation sequencing technologies have allowed rapid high-efficiency sequencing of the exome, the entire coding region, called whole exome sequencing (WES), and of the entire genome, as in whole genome sequencing (WGS). WES combines the utility of detailing the entire coding portion of the genome that harbors ~ 85% of known disease mutations, and the feasibility of result interpretation at a lower cost as compared to WGS [11]. Currently, WES is a commonly used genomic approach in both clinical and research settings. Although both clinical and research labs may employ WES, data analysis and interpretation usually differ between these settings. While research testing may explore novel genotype-phenotype correlations, clinical labs usually use a focused analysis approach looking only at genes known to associate with a particular phenotype. A focused approach to a WES comprehensive dataset is readily useful clinically and, importantly, enables future reanalysis of additional disease-causing genes identified over time. WES dataset analysis also has the advantage over limited gene panel testing of allowing to assess comprehensive phenotypes, including all clinical manifestations of a disease condition rather than a single clinical diagnosis [12]. Such WES analysis requires that clinicians submit all relevant phenotypic information to the genetic testing lab rather than providing a single clinical or biopsy diagnosis. Large databases of comprehensive genotype information linked to detailed phenotypes archived in the electronic health records should be viewed as valuable datasets that can be mined over time for relevant information, which might inform clinical decision-making as knowledge of specific conditions evolve. The perceived advantage of WGS over WES is to enable examination of biologically relevant non-coding variation especially for genetically unsolved cases. This information is currently used as research tool but will likely become more available, interpretable, and cost-effective in the near future.

## Case-based genetic testing

We illustrate the most applicable genetic testing approach for actual clinical scenarios.

### Example 1: a kindred with syndromic FSGS

**Fig. 1:**
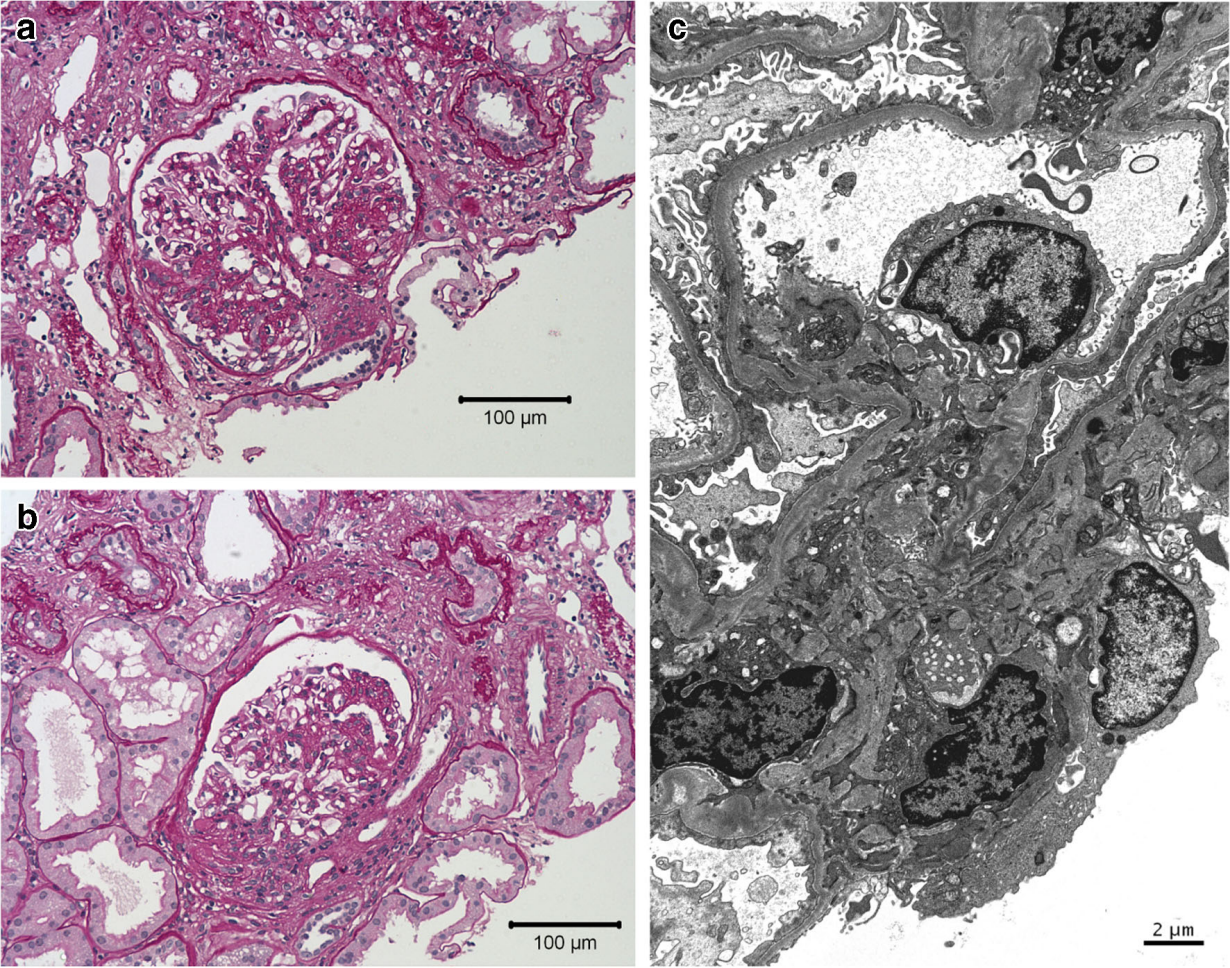
Light (**a**, **b**) and electron microscopy (**c**) of a kidney biopsy specimen showing focal segmental glomerulosclerosis in the 22-year-old father with clinically unsuspected branchio-oto-renal syndrome (BOR1) carrying *EYA1* mutation. Light microscopy showed 3 globally sclerotic glomeruli and 3 glomeruli with perihilar focal segmental glomerulosclerosis and glomerulomegaly (**a**), foam cells, dysplastic tubules, tubular atrophy, interstitial fibrosis involving > 60% of cortex (**b**); electron microscopy showed foot process effacement without GBM lamination (**c**) and negative immunofluorescence lead to a diagnosis of FSGS

An 8-month-old male with history of maternal oligohydramnios and a failed neonatal hearing test had a physical exam notable for left plagiocephaly, low set ears with bilateral preauricular pits, asymmetric nasal bridge, right torticollis, and bilateral cryptorchidism. No branchial fistulae were noted. Ultrasound showed bilateral small echogenic kidneys with poor corticomedullary differentiation and normal intra-abdominal testes. His serum creatinine was 0.6 mg/dL and a VCUG was normal. WES performed as a clinical test with the presumed diagnosis of branchio-oto-renal syndrome (BOR) identified a heterozygous mutation in *EYA1* (eyes absent transcriptional coactivator and phosphatase 1), a transcriptional factor that plays a role in development of the kidney, eye, ear, and branchial arches. The *EYA1* mutation (c.1748T>C: p. Leu583Pro) was consistent with the diagnosis of BOR (OMIM 113650) [13]. Family history revealed biopsy-proven FSGS (Fig. 1) at age 22 years in the patient’s father, who had been evaluated for nephrotic range proteinuria up to 2.6 g/day, microscopic hematuria and elevated serum creatinine (2.9 mg/dL), negative autoimmune serologies and long-standing hearing loss. Physical examination on the father revealed a branchial fistula, and genetic testing using Sanger sequencing showed the same *EYA1* mutation. An identical *EYA1* mutation was also confirmed in a younger male sibling with bilateral preauricular pits, abnormal pinnae, and a neck brachial cleft sinus.

#### Messages

WES is a comprehensive genomic approach that enables to resolve the genetic etiology of rare syndromic presentations. The *EYA* mutation identified by WES confirmed the clinical suspicion of BOR in the 8-month-old proband. Subsequent, direct sequencing of the specific mutation as part of cascade genetic testing was useful to test affected family members. The proband’s father illustrates that BOR syndrome can present as FSGS with autosomal dominant (AD) inheritance and clinically missed syndromic features. Syndromic diagnoses can be elusive due to varying clinical presentations within the same family, which may result from variable penetrance of the underlying mutation or from other modifier mutation effect. Consistent with this argument of modifier mutations, a retrospective analysis of a large series of 2076 patients carrying a molecular diagnosis identified ~ 5% patients with more than one genetic diagnoses, often involving genes in the same molecular pathway [14].

Establishing a genetic diagnosis in syndromic FSGS facilitates management, including avoidance of toxic immunosuppressive therapy and informs post-transplant recurrence risk and donor selection, as children with FSGS due to genetic mutations are less likely to respond to glucocorticoid therapy and also less likely to have disease recurrence after kidney transplantation [15]. Syndromic FSGS may benefit from early recognition, for example, molecular diagnosis in FSGS as part of Frasier syndrome can benefit from monitoring for Wilms’ tumor and gonadoblastoma [6, 16]. The most direct example of a molecular diagnosis affecting therapy choice in FSGS is that patients with mutations in the genes of coenzyme Q10 biosynthesis pathway that lead to FSGS may benefit from coenzyme Q10 supplementation in terms of reduction in proteinuria [17]. Similarly, the identification of *APOL1* risk alleles that confer FSGS susceptibility in African-Americans can be done using targeted or comprehensive sequencing approaches, though this finding informs disease understanding, it may have limited clinical implications to date [18, 19].

### Example 2: Sporadic FSGS undergoing pre-transplant evaluation

A 16-year-old girl with ESRD secondary to steroid-resistant nephrotic syndrome diagnosed at 10 years of age and unresponsive to immunosuppressive medications was evaluated for a live-related kidney transplant from her apparently healthy mother. The proband’s kidney biopsy had shown FSGS, and results of a FSGS gene panel performed by a CLIA-certified laboratory employing Sanger sequencing for four genes commonly mutated in childhood FSGS reported a heterozygous mutation in *NPHS2* (p. Ala284Val) as a rare missense variant (one allele present in the general population gnomAD database (https://gnomad.broadinstitute.org) out of 249,998 total tested, i.e., allele frequency of 4 × 10^−6^). Homozygous or bi-allelic mutations in *NPHS2* are the most common cause of autosomal recessive (AR) childhood FSGS [20]. However, no pathogenic mutation or other variant of interest was reported in *NPHS2* or the other three FSGS genes sequenced at the time by the commercial laboratory. In 2014, Tory et al. showed that the pathogenicity of the *NPHS2* Arg229Gln variant is dependent on the presence of specific *NPHS2* mutations *in trans*, the *NPHS2* Ala284Val variant being one of them [21]. A careful assessment of the patient’s heterozygous *NPHS2* mutation during her pre-transplant evaluation years later suggested that the patient may also carry the Arg229Gln variant. Upon request, the commercial laboratory reported this was the case, thus confirming the bi-allelic mode of inheritance of *NPHS2-*mediated AR FSGS*.* The Arg229Gln variant (rs61747728; allele frequency of 3%; with 8446 alleles reported out of 244,704 total tested in the gnomAD database) is a common *NPHS2* allele, thus had been deemed a polymorphism and was not reported by the commercial laboratory in the first place.

#### Messages

Gene panel–based genetic testing for a specific phenotype is ever evolving, because novel causative genes are constantly added to the diagnostic list and also variants previously considered benign are shown to be of significance. In this case, knowledge that specific podocin (*NPHS2*) mutations *in trans* with the Arg229Gln polymorphism can be disease causing leading to further clarification of the genetic results [21]. This complete information helped to confirm the causative disease inheritance and assess donor suitability. Genetic testing for FSGS that aims to identify variants in known genes can be achieved by using gene panel–based genetic testing, whereas identification of novel genes or novel variant association as causative requires unbiased and comprehensive genome (WGS) or exome-scale sequencing (WES).

### Example 3: Collagen IV mutations and histopathological FSGS presentation

A 59-year-old female with stage 5 CKD due to FSGS on kidney biopsy and long-standing hypertension underwent genetic evaluation during workup for a live-related kidney transplant. The donor, her 62-year-old female sibling, had normal kidney function and history of persistent microscopic hematuria. No hearing loss or ocular abnormalities were reported in either of them. The patient WES identified a novel heterozygous loss of function *COL4A4* variant (NM_000092: exon 39: c.3704delC: p. P1235fs), deemed likely pathogenic since the deletion of a single base pair at this position leads to a frameshift mutation resulting in a premature stop codon after 53 novel amino acids are added to the protein sequence. No mutation was identified in a panel of known FSGS genes. These genetic results are consistent with the presence of a likely pathogenic heterozygous variant in *COL4A4*, an Alport syndrome gene; however, in the absence of clinical features of Alport syndrome, the term “autosomal dominant Alport syndrome” should be avoided as discussed in a recent consensus statement [22]. Given the history of microscopic hematuria, the sibling was evaluated using an Alport syndrome gene panel performed by a commercial lab, before being cleared as a kidney donor. No pathogenic variant was found in the *COL4A3*, *COL4A4*, or *COL4A5* genes in the sibling.

#### Messages

Comprehensive genetic testing such as WES can help clarify etiological misclassifications and overlapping diagnoses in glomerular disorders. Genetic testing in the presented case enabled to avoid a kidney biopsy for donor evaluation. It is possible that an undetected genetic modifier or non-genetic factors such as presence of hypertension resulted in CKD in this presented patient with a heterozygous pathogenic *COL4A4* variant. Mutations in *COL4A5*, *COL4A3*, and *COL4A4*, the three Alport syndrome genes, have been reported in patients presenting as FSGS [23, 24]. The genetic diagnosis is important for therapy, outcome prediction, and for live donor pre-transplant evaluation. While pathogenic *COL4A5* variants cause x-linked Alport syndrome and bi-allelic mutations in *COL4A3* or *COL4A4* cause the autosomal recessive form, the interpretation of the biological significance of rare pathogenic but heterozygous *COL4A3* or *COL4A4* mutations can be challenging [22]. WES enables detection of co-existent mutations in more than one gene, which may be missed by gene panel–based testing. The finding of rare collagen IV mutations deemed pathogenic by bioinformatics prediction scores and detected to co-exist with other disease-causing FSGS gene variants intriguingly argues for possible digenic inheritance or a gene modifier role for these mutations as an interesting observation that may merit systematic investigation [25, 26]. Similarly, the finding of mutations in another collagen IV gene coding for the α1 chain of type IV collagen (*COL4A1*) that cause HANAC syndrome (*h*ereditary *a*ngiopathy, *n*ephropathy, *a*neurysms, and muscle *c*ramps) may present a diagnostic conundrum, given that its renal manifestations may include microscopic hematuria and cystic kidney disease [27, 28].

### Example 4: Complement-mediated glomerular disease—familial atypical HUS

A 9-year-old previously healthy female of mixed Caucasian ethnicity presented with non-immune hemolytic anemia, thrombocytopenia, acute kidney injury, and lethargy, without recent history of infection or diarrhea. Stool was negative for Shiga toxin–producing *E. coli*. Low serum C3 was consistent with alternate complement pathway activation. WES identified a rare heterozygous *CFI* (complement factor I) mutation (NM_000204: exon1: c.1A>G: p. M1V) that disrupts the start codon, leading to loss of function or a new translation initiation site and a new reading frame. The patient’s mother, who was subsequently confirmed to carry the same *CFI* mutation, has had two episodes of atypical hemolytic uremic syndrome (aHUS) during pregnancy leading to ESRD, and a brief renal allograft recurrence of HUS.

#### Messages

The natural history and clinical course of aHUS in the native and transplant kidney relates to the underlying genetic mutation. Inherited or acquired deficiencies of the complement regulatory system account for up to 60% of etiologically defined aHUS with mutations mostly in heterozygosis reported in complement regulatory genes factor H (*CFH*), *CFI*, membrane cofactor protein, thrombomodulin, factor B, *C3*, or presence of autoantibodies to CFH [29]. Mutations causing aHUS have also been reported in *MMACHC* and *MTRR* genes of the cobalamin pathway and the lipid kinase diacylglycerol kinase epsilon (*DGKE*) gene [30]. Gene panel–based testing can identify the causative mutation and at-risk haplotypes and aid diagnosis in about 60% cases.

### Example 5: Variants of uncertain significance—classification based on ACMG criteria

A 17-year-old African-American male with long-standing near nephrotic range proteinuria had a kidney biopsy showing FSGS. His family history revealed that his mother developed ESRD of unclear etiology at 56 years of age. No other comorbidity or hearing loss was reported in either of them. Genetic evaluation for familial CKD using WES in the proband identified a novel *GATA3* missense variant (GATA Binding Protein 3 transcription factor), which leads to substitution of a highly conserved amino acid residue (*GATA3*: NM_002051: cC703A:pP235T) predicted to be deleterious by bioinformatics scores. No mutation was detected by the clinical lab in genes known to cause FSGS, nephrotic syndrome, or CKD, including absence of *APOL1* risk alleles. Genetic testing could not be done for the mother.

#### Messages

A novel heterozygous missense *GATA3* variant of uncertain significance (VUS) was identified by WES in a FSGS patient. Previously reported heterozygous missense *GATA3* variants cause HRD (hypoparathyroidism, sensorineural deafness, and renal dysplasia) syndrome, which has varied presentations including a kidney-limited phenotype [31, 32]. For clinical reporting of genetic data, gene variants are classified as pathogenic, likely pathogenic, or VUS based on current knowledge [33]. In the absence of known direct disease association, variants classified as VUS should not be used for clinical decision-making [33]. However, variant classification and interpretation may change over time and re-interpretation of WES data may be useful and necessary. Evaluation using a known FSGS gene panel would have missed this novel *GATA3* mutation that may have led to a kidney-limited phenotype of HRD, as previously recognized [32], possibly resulting in FSGS secondary to renal dysplasia. Interpretation of novel and incidental findings identified by WES can be challenging, and requires proof of causality such as co-segregation of mutation with disease within affected families, experimental models replicating the phenotype, which often take extended time periods. The genetic and biological validation of novel genes or novel functions of known genes remains the biggest obstacle for WES data interpretation.

### Insights and questions

Suspected inherited glomerular diseases involving defects in the GBM, the podocyte, or endothelial damage leading to thrombotic microangiopathy can result in a molecular diagnosis in 30–60% of cases depending on the age at disease onset, with higher diagnostic rates in familial cases [34]. For example, mutations of podocyte-associated genes account for approximately 30% of pediatric cases of steroid-resistant nephrotic syndrome with one of the four genes (*NPHS1*, *NPHS2*, *WT1*, and *LAMB2*) identified in 66% of nephrotic syndrome manifesting within the first year of life [35]. Similarly, known complement genes account for ~ 60% of cases in large cohorts of atypical hemolytic uremic syndrome [29]. While there is a broad availability of genetic testing approaches for clinical and research use, the gene panel–based approach is most frequently reported. Using a targeted next-generation sequencing of 140 selected genes, approximately one-third of the 98 cases with suspected inherited glomerular diseases remained without any identified genetic cause [34].

Comprehensive sequencing using WES or WGS has the potential to identify novel genes and novel presentations of known genes *in addition to* identifying known genetic causes albeit the challenge of genetic validation and biological interpretation. The clinical importance of such comprehensive genomic sequencing in infant and pediatric conditions has been described in the form of retrospective data, mostly case reports showing a diagnostic yield advantage in one-third of the cases investigated, with higher yield when patient-parent trios are evaluated [36]. The limited information available on clinical utility or economic impact of clinical genomic sequencing supports a favorable effect [34, 36]. The use of a comprehensive 140 gene panel for suspected inherited glomerular diseases showed maximum clinical utility in familial cases and those with a lower age at onset or atypical clinical presentations. An accurate genetic cause as a new or corrected diagnosis was established in 17% cases in this series of genetic kidney conditions [34].

**Fig. 2:**
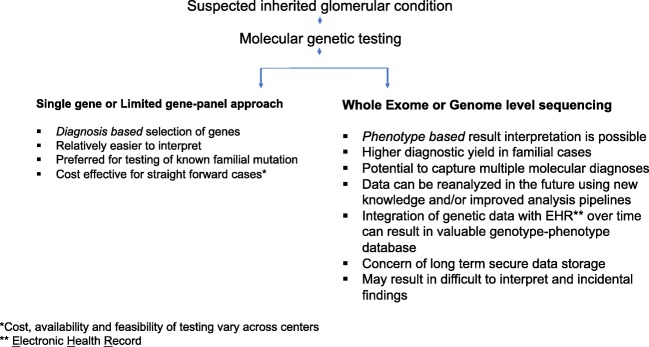
Inherited glomerulopathies and choices for genetic testing

Genome-wide or exome-wide sequencing combines the advantage of analyzing a large pool of genes based on current knowledge and the option of revisiting this analysis as more information becomes available over time (Fig. 2). This is congenial to the expectation that the list of monogenic causes for glomerular disease is ever evolving and also that some cases may merit investigation for possible oligogenic involvement or complex inheritance [25, 26]. Cost considerations and the challenge of massive data storage can limit the use of available wide-scale genomic approaches. However, this cost may offset the expense of repeat testing in the future. Such approach also provides the opportunity of linking genetic data to individual electronic medical records resulting in large genotype-phenotype databases that can be queried for relevant information. It is also likely that comprehensive genomic approaches will identify non-monogenic causes for previously unclassified conditions in the near future. The challenges of result turn-around time and data interpretation including VUS and incidental findings are expected to decrease with greater usage.

## Conclusions

Familial and inherited glomerular diseases constitute a high-yield group where advances in genetic diagnostic methods can be efficiently utilized to achieve favorable clinical outcomes and pursue opportunities for research. Categorization based on the pre-test probability of a genetic diagnosis and selection of the most appropriate genetic testing method can maximize feasibility of a clinically meaningful result interpretation. Greater use of comprehensive genomic testing methods will create databases linking genotype-phenotype information that will serve to overcome challenges of data interpretation in the future and contribute to powerful utilization of genomic innovations. On the research front, optimization of knock-in gene editing models and relevant functional assays, for example, using human podocyte cell lines, might facilitate testing of multiple gene variants for their potential disease-causing effect.

## Questions: (answers appear after the reference list)

Question 1:

Choose the factor(s) likely to improve the yield and clinical usefulness of a genetic evaluation such as a genetic panel or whole exome sequencing (WES):

A. A high suspicion of a genetic condition based on the clinical presentation and family history obtained by the ordering physician
B. Detailed phenotype information made available to the genetic testing laboratory
C. Availability of DNA from parents or other affected family members
D. All the above

Question 2:

Whole Exome Sequencing results reported by a clinical laboratory may miss which of the following types of genetic variants

A. A large structural DNA variation or Copy Number Variation
B. Deep intronic variants
C. Common variants in complex disease genes
D. Variants in genes not known to be causative of the clinical phenotype at the time of the study.
E. All the above

Question 3:

A 14 years old male has nephrotic range proteinuria and CKD, mild craniofacial asymmetry and hearing loss, his renal biopsy shows FSGS. Genetic evaluation may reveal:

A. *COL4A5* mutation (X-linked Alport syndrome)
B. *EYA1* heterozygous mutation (BOR syndrome)
C. *COL4A4* heterozygous mutation (*COL4*-associated FSGS)
D. *NPHS2* mutation (AR FSGS)
E. A) and B)

Question 4:

What genetic evaluation approach would be most suitable to obtain a genetic diagnosis in the patient described above?

A. Karyotype
B. WES
C. Gene panel
D. WGS

Question 5:

A 19 years old female has borderline hypertension and a history of hematuria and low-grade proteinuria. Multiple family members have microscopic hematuria and/or renal insufficiency. You consider ordering either genetic testing of *COL4A3*, *COL4A4*, and *COL4A5*, which consists of sequencing and deletion testing for the 3 genes, or WES for microscopic hematuria. What are the likely benefits of WES in this case?

A. Identification of a variant of unknown significance (VUS) in *COL4A3*, *COL4A4*, or *COL4A5*
B. Detection of a deletion mutation in *COL4A3, COL4A4, or COL4A5*
C. Identification of a mutation in the coding sequence of another gene responsible for microscopic hematuria
D. Coverage by insurance
E. Reveal epigenetic factors leading to disease variability.
